# Supplementary material for: Health-Related Quality of Life by Gulf War Illness Case Status
Source: Int J Environ Res Public Health. 2022 Apr 7;19(8):4425. doi: 10.3390/ijerph19084425 (PMC9026791; doi:10.3390/ijerph19084425)
Supplement: Supplementary file 1 [file ijerph-19-04425-s001.zip › ijerph-1585402-supplementary.pdf]

International Journal of
Environmental Research and Public Health

MDPI

Figure S1. Sample Flow.

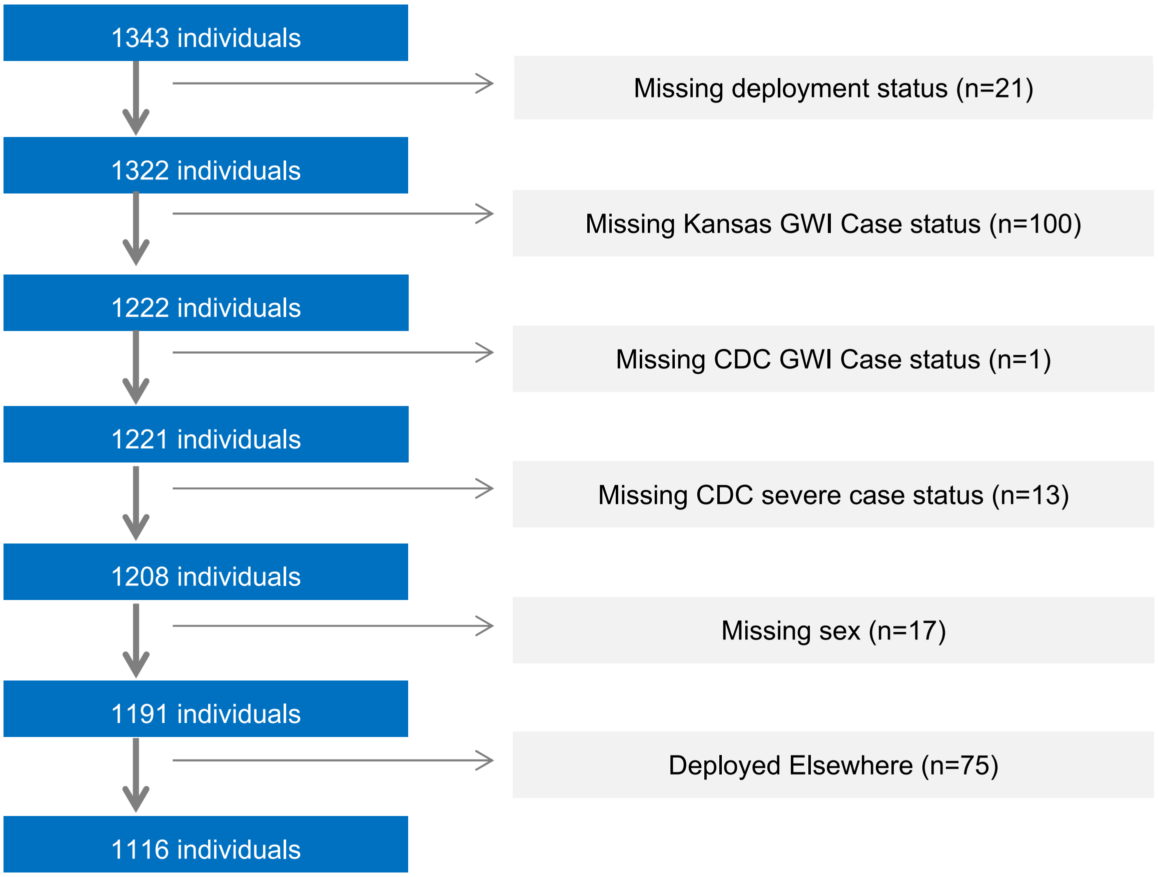

Reprinted from Life Sci, vol(278), Gulf War illness in the Gulf War Era Cohort and Biorepository: The Kansas and Centers for Disease Control definitions, Gifford, E.J., Vahey, J., Hauser, E.R., Sims, K.J., Efird, J.T., Dursa, E.K., Steele, L., Helmer, D.A. Provenzale, D., page no. 119454--, Copyright (2021), with permission from Elsevier.

Table S1. Symptoms in the Kansas Gulf War Illness Definition.

Domain	Symptom
Fatigue	Fatigue
	Feeling unwell after exercise
	Difficulty getting to or staying asleep
	Not feeling rested after sleep
Pain	Pain in joints
	Pain in muscles
	Body pain where you hurt all over
Neurological, Mood, or Cognitive	Difficulty remembering recent information
	Feeling irritable or having angry outbursts
	Numbness or tingling in extremities
	Headaches
	Eyes very sensitive to light
	Trouble finding words when speaking
	Feeling down or depressed
	Difficulty concentrating

	Night sweats<br>Feeling dizzy, lightheaded, or faint<br>Low tolerance for heat or cold<br>Symptoms in response to smells or chemicals<br>Blurred or double vision<br>Tremors or shaking
Gastro-intestinal	Diarrhea<br>Nausea or upset stomach<br>Abdominal pain or cramping
Respiratory	Difficulty breathing<br>Frequent coughing without a cold<br>Wheezing in chest
Skin	Skin rash<br>Other skin problems

Table S2. Symptoms in the CDC GWI Case Definition.

Symptom Domain	Symptom
Fatigue	Fatigue
Musculoskeletal	Pain in joints<br>Pain in muscles<br>Stiffness in joints
Mood–Cognition	Difficulty remembering recent information or difficulty concentrating [1]<br>Difficulty remembering recent information<br>Difficulty concentrating<br>Trouble finding words when speaking<br>Feeling moody<br>Feeling down or depressed<br>Difficulty getting to or staying asleep[1]<br>Feeling anxious

[1] The CDC GWI definition originally asked about Difficulty remembering recent information or difficulty concentrating. However, the GWEB included two separate items Difficulty remembering recent information and Difficulty concentrating. Responses were combined into 1 score. CDC = Centers for Disease Control and Prevention

Table S3. Crosstabulation of Gulf War illness case status by Case Definitions.

	**Centers for Disease Control**						**Total**	
	**Severe GWI**		**GWI (Not Severe)**		**Not GWI**			
**Kansas Category**	***n***	**%**	***n***	**%**	***n***	**%**	**N**	**%**
KS Sym+/Dx−	117	44.3	NS		Sup.		426	38.2
KS Sym+/Dx+	145	54.9	NS		Sup.		345	30.9
KS Sym−/Dx+	NS		NS		48	25.7	106	9.5
KS Sym−/Dx−	Sup.		NS		121	64.7	239	21.4
Total	264	100	665	100	187	100	1116	100

Note: Sym = symptom; Dx = diagnosis; CDC = Centers for Disease Control and Prevention; KS = Kansas; GWI = Gulf War illness. Sup. = suppressed cell size <11; NS = Not shown due to the disclosure concerns given the need to suppress cells with fewer than 11 observations.

Table S4. Multivariable regressions of Health-Related Outcomes by Kansas Gulf War Illness Status.

Covariates	HRQOL		Other Indices of Health			
	Physical	Mental	Sleep Disturbance	PTSD	Depression	Pain
	Beta (s.e.)	Beta (s.e.)	Beta (s.e.)	Beta (s.e.)	Beta (s.e.)	Beta (s.e.)
KS GWI status (ref: GWI)						
Sym+/Dx+	−4.583 [a]	−3.923 [a]	1.367 [c]	7.100 [a]	2.357 [a]	0.585 [b]
	(0.826)	(0.903)	(0.654)	(1.204)	(0.438)	(0.186)
Sym−/Dx+	6.580 [a]	8.903 [a]	−10.23 [a]	−12.66 [a]	−4.691 [a]	−2.180 [a]
	(1.147)	(1.005)	(0.926)	(1.037)	(0.379)	(0.271)
Sym−/Dx−	10.74 [a]	8.210 [a]	−11.20 [a]	−12.44 [a]	−5.069 [a]	−2.620 [a]
	(0.720)	(0.781)	(0.688)	(0.888)	(0.320)	(0.175)
Deployed (ref: not deployed)	−1.060	−2.648 [a]	2.204 [a]	4.445 [a]	1.458 [a]	0.399 [c]
	(0.713)	(0.730)	(0.596)	(0.950)	(0.334)	(0.165)
Age (at survey)	−0.125 [b]	0.271 [a]	−0.160 [a]	−0.306 [a]	−0.102 [a]	−0.005
	(0.0394)	(0.0420)	(0.0335)	(0.0509)	(0.0191)	(0.010)
Female (ref: male)	−1.085	−2.217 [b]	−0.225	0.614	0.823 [c]	0.332
	(0.770)	(0.847)	(0.625)	(1.067)	(0.389)	(0.174)
Race/ethnicity (ref: White, non-Hispanic)						
Black, non-Hispanic	−1.726 [c]	−2.020 [c]	0.536	4.734 [a]	0.673	0.983 [a]
	(0.854)	(0.938)	(0.689)	(1.257)	(0.433)	(0.207)
Hispanic	−1.179	−2.774 [c]	0.956	5.241 [a]	1.569 [b]	0.712 [c]
	(1.125)	(1.186)	(0.858)	(1.569)	(0.579)	(0.289)
Other	−2.573 [c]	−2.706	2.140	7.347 [a]	1.898 [c]	0.981 [b]
	(1.237)	(1.586)	(1.107)	(2.183)	(0.805)	(0.305)
Missing or Unknown	−1.435	−6.888 [a]	−2.416	9.757 [c]	2.324	0.698
	(2.113)	(1.961)	(1.850)	(3.796)	(1.291)	(0.526)
Income (ref: $100,000+)						
<$30,000	−4.445 [a]	−7.513 [a]	3.233 [a]	9.383 [a]	3.247 [a]	0.925 [a]
	(1.188)	(1.282)	(0.957)	(1.711)	(0.594)	(0.271)
$30,000–$59,999	−2.193 [c]	−3.707 [a]	1.584 [c]	5.925 [a]	1.918 [a]	0.621 [b]
	(0.918)	(0.977)	(0.759)	(1.261)	(0.449)	(0.214)
$60,000–$99,999	−1.136	−2.472 [b]	0.877	2.694 [b]	1.068 [b]	0.171
	(0.789)	(0.853)	(0.662)	(1.009)	(0.367)	(0.183)
Unknown	−3.527 [b]	−2.371	1.258	2.970	1.209	0.312
	(1.360)	(1.610)	(1.024)	(1.906)	(0.693)	(0.327)
Education (ref: Master's, Professional, or Doctorate's degree)						
<Associates degree	−2.947 [c]	2.011	−0.209	−0.222	−0.578	−0.005
	(1.323)	(1.461)	(1.082)	(1.954)	(0.672)	(0.322)
Bachelor's or Associate's degree	−1.805 [c]	1.103	0.945	−0.625	0.118	0.220
	(0.734)	(0.851)	(0.657)	(1.013)	(0.373)	(0.182)
Unknown	−1.726	1.430	−0.207	2.233	0.0216	0.601
	(1.985)	(2.576)	(2.054)	(3.409)	(1.358)	(0.560)

Military Component (ref: Active Duty)						
Active Duty & Reserves	1.032	−0.407	−1.188	0.594	−0.361	−0.066
	(0.796)	(0.829)	(0.666)	(1.083)	(0.389)	(0.178)
Reserves	2.755 [b]	0.648	−1.037	−2.550 [c]	−0.956 [c]	−0.180
	(0.846)	(0.877)	(0.710)	(1.078)	(0.387)	(0.204)
Military Branch (ref: Army)						
Navy	2.245 [b]	0.0293	−1.399	−1.906	−0.401	−0.428 [c]
	(0.870)	(1.005)	(0.745)	(1.172)	(0.432)	(0.203)
Airforce	3.333 [b]	−0.666	−0.902	−3.004 [c]	−0.329	−0.799 [b]
	(1.012)	(1.043)	(0.841)	(1.338)	(0.481)	(0.245)
Marines	2.309 [c]	−0.954	−0.468	−0.947	−0.323	−0.354
	(0.956)	(1.052)	(0.872)	(1.266)	(0.470)	(0.234)
National Guard	0.669	−2.147	0.290	0.615	0.646	−0.031
	(1.094)	(1.165)	(0.908)	(1.599)	(0.568)	(0.258)
Other	−0.195	−3.555 [c]	2.297 [c]	2.647	0.965	0.452
	(1.427)	(1.569)	(1.121)	(2.095)	(0.795)	(0.321)
Constant	49.80 [a]	35.85 [a]	62.76 [a]	45.37 [a]	10.40 [a]	3.826 [a]
	(2.313)	(2.574)	(2.007)	(3.021)	(1.147)	(0.567)
Observations	1109	1106	1076	1096	1096	1100
R-squared	0.342	0.298	0.388	0.397	0.383	0.342

Note: a $p < 0.001$, b $p < 0.01$, c $p < 0.05$. s.e. = robust standard error; Sym = symptom; Dx = Diagnosis.

Table S5. Multivariable regressions of Health-Related Outcomes by CDC Gulf War Illness Status.

Covariates	HRQOL		Other Indices of Health			
	Physical	Mental	Sleep Disturbance	PTSD	Depression	Pain
	Beta (s.e.)	Beta (s.e.)	Beta (s.e.)	Beta (s.e.)	Beta (s.e.)	Beta (s.e.)
CDC GWI status (ref: CDC severe)						
CDC mild-to-moderate GWI	12.59 [a]	12.07 [a]	−9.854 [a]	−21.85 [a]	−7.896 [a]	−2.685 [a]
	(0.786)	(0.856)	(0.621)	(1.093)	(0.390)	(0.178)
Noncase	19.42 [a]	19.50 [a]	−19.26 [a]	−31.48 [a]	−11.81 [a]	−4.757 [a]
	(0.946)	(0.937)	(0.822)	(1.155)	(0.404)	(0.220)
Deployed (ref: not deployed)	−0.403	−1.886 [b]	1.876 [b]	2.966 [a]	0.924 [b]	0.270
	(0.717)	(0.700)	(0.593)	(0.822)	(0.303)	(0.161)
Age (at survey)	−0.204 [a]	0.214 [a]	−0.133 [a]	−0.204 [a]	−0.0630 [a]	0.007
	(0.039)	(0.038)	(0.031)	(0.043)	(0.016)	(0.009)
Female (ref: male)	−0.163	−1.151	−1.113	−1.319	0.210	0.108
	(0.753)	(0.826)	(0.620)	(0.981)	(0.360)	(0.166)
Race/ethnicity (ref: White, non-Hispanic)						
Black, non-Hispanic	−1.439	−1.550	0.245	3.684 b	0.364	0.903 [a]
	(0.816)	(0.900)	(0.692)	(1.157)	(0.388)	(0.203)
Hispanic	0.760	−0.669	−0.507	1.126	0.153	0.305
	(1.064)	(1.069)	(0.841)	(1.240)	(0.481)	(0.270)

Other	−0.161	−0.290	0.150	2.928	0.373	0.440
	(1.182)	(1.319)	(0.947)	(1.656)	(0.618)	(0.295)
Missing or Unknown	−1.675	−6.655 [a]	−2.256	8.393 [c]	2.266	0.713
	(1.977)	(1.821)	(1.599)	(3.585)	(1.276)	(0.475)
Income (ref: $100,000+)						
<$30,000	−4.333 [a]	−7.174 [a]	3.235 [a]	8.423 [a]	2.910 [a]	0.881 [a]
	(1.153)	(1.254)	(0.916)	(1.436)	(0.535)	(0.253)
$30,000-$59,999	−2.489 [b]	−3.636 [a]	1.607 [c]	5.734 [a]	1.822 [a]	0.605 [b]
	(0.869)	(0.981)	(0.713)	(1.166)	(0.407)	(0.203)
$60,000-$99,999	−0.801	−1.908 [c]	0.228	1.795 [c]	0.735 [c]	0.023
	(0.762)	(0.808)	(0.661)	(0.910)	(0.319)	(0.176)
Unknown	−3.115 [c]	−1.722	0.610	1.890	0.819	0.154
	(1.417)	(1.379)	(1.015)	(1.562)	(0.581)	(0.301)
Education (ref: Master's, Professional, or Doctorate's degree)						
<Associate's degree	−3.724 [b]	1.725	0.148	0.442	−0.298	0.0908
	(1.274)	(1.350)	(0.987)	(1.695)	(0.560)	(0.315)
Bachelor's or Associate's degree	−1.971 [b]	1.229	0.862	−0.859	0.0780	0.240
	(0.746)	(0.791)	(0.632)	(0.872)	(0.314)	(0.180)
Unknown	−2.052	1.331	0.185	1.955	0.287	0.650
	(1.899)	(2.132)	(1.871)	(2.721)	(1.316)	(0.497)
Military Component (ref: Active Duty)						
Active Duty & Reserves	1.255	−0.310	−1.194	0.277	−0.436	−0.086
	(0.762)	(0.758)	(0.622)	(0.925)	(0.324)	(0.170)
Reserves	2.454 [b]	0.114	−0.717	−1.609	−0.630	−0.091
	(0.849)	(0.904)	(0.671)	(1.032)	(0.370)	(0.200)
Military Branch (ref: Army)						
Navy	2.084 [c]	−0.334	−1.048	−1.314	−0.155	−0.346
	(0.827)	(0.941)	(0.732)	(1.031)	(0.374)	(0.196)
Airforce	2.611 [b]	−1.426	−0.132	−1.743	0.147	−0.613 [b]
	(0.974)	(0.997)	(0.804)	(1.084)	(0.426)	(0.229)
Marines	2.420 c	−0.943	−0.150	−0.845	−0.230	−0.341
	(0.961)	(0.970)	(0.794)	(1.138)	(0.370)	(0.229)
National Guard	0.314	−2.489 [c]	0.809	1.100	0.863	0.063
	(1.066)	(1.115)	(0.861)	(1.352)	(0.498)	(0.235)
Other	−0.195	−3.586 [b]	2.261 [c]	2.989	1.057	0.426
	(1.331)	(1.330)	(1.074)	(1.671)	(0.629)	(0.291)
Constant	44.03 [a]	28.51 [a]	68.18 [a]	59.16 [a]	15.03 [a]	5.183 [a]
	(2.399)	(2.427)	(1.983)	(2.805)	(1.028)	(0.547)
Observations	1109	1106	1076	1096	1096	1100
R-squared	0.382	0.377	0.444	0.540	0.529	0.403

Note: a $p < 0.001$, b $p < 0.01$, c $p < 0.05$. s.e. = robust standard error; Sym = symptom; Dx = Diagnosis; ref = omitted reference category.

Table S6. Linear Combination Tests of Regression Coefficients for Gulf War Case Status Subtypes

Kansas (Supplements Table A3)			CDC (Supplements Table A4)		
**Dependent Variable**	***p*-Value**	**F or chi2**		***p*-Value**	**F or chi2**
Physical component score			Physical component score		
KS noncase (1): Sym+/Dx+ = KS noncase (2): Sym−/Dx+	<0.001	86.872	GWI mild-to-moderate = Noncase	<0.001	88.504
KS noncase (1): Sym+/Dx+ = KS noncase (3): Sym−/Dx−	<0.001	349.619			
KS noncase (2): Sym−/Dx+ = KS noncase (3): Sym−/Dx−	<0.001	14.553			
Mental compoinent score			Mental component score		
KS noncase (1): Sym+/Dx+ = KS noncase (2): Sym−/Dx+	<0.001	147.975	GWI mild-to-moderate = Noncase	<0.001	129.507
KS noncase (1): Sym+/Dx+ = KS noncase (3): Sym−/Dx−	<0.001	184.702			
KS noncase (2): Sym−/Dx+ = KS noncase (3): Sym−/Dx−	0.454	0.561			
KS noncase (1): Sym+/Dx+ = KS noncase (2): Sym−/Dx+	0.213	1.556			
Sleep			Sleep		
KS noncase (1): Sym+/Dx+ = KS noncase (2): Sym−/Dx+	<0.001	155.810	GWI mild-to-moderate = Noncase	<0.001	207.884
KS noncase (1): Sym+/Dx+ = KS noncase (3): Sym−/Dx−	<0.001	284.561			
KS noncase (2): Sym−/Dx+ = KS noncase (3): Sym−/Dx−	0.298	1.082			
PTSD			PTSD		
KS noncase (1): Sym+/Dx+ = KS noncase (2): Sym−/Dx+	<0.001	287.289	GWI mild-to-moderate = Noncase	<0.001	196.831
KS noncase (1): Sym+/Dx+ = KS noncase (3): Sym−/Dx−	<0.001	313.586			
KS noncase (2): Sym−/Dx+ = KS noncase (3): Sym−/Dx−	0.796	0.067			
Depression			Depression		
KS noncase (1): Sym+/Dx+ = KS noncase (2): Sym−/Dx+	<0.001	280.703	GWI mild-to-moderate = Noncase	<0.001	278.111
KS noncase (1): Sym+/Dx+ = KS noncase (3): Sym−/Dx−	<0.001	358.574			
KS noncase (2): Sym−/Dx+ = KS noncase (3): Sym−/Dx−	0.213	1.556			
Pain			Pain		
KS noncase (1): Sym+/Dx+ = KS noncase (2): Sym−/Dx+	<0.001	97.276	GWI mild-to-moderate = Noncase	<0.001	144.648
KS noncase (1): Sym+/Dx+ = KS noncase (3): Sym−/Dx−	<0.001	268.0571			
KS noncase (2): Sym−/Dx+ = KS noncase (3): Sym−/Dx−	0.0977	2.747			

Note: Sym = symptom; Dx = diagnosis.

Table S7. Proportion of GWECB Veterans meeting Depression, Post-Traumatic Stress, and Pain cutoff points by GWI Case Status.

**Outcome**		**Kansas**								**CDC**					
	**Total**	**KS GWI: Sym+/Dx− (*n* = 426)**		**KS noncase (1): Sym+/Dx+ (*n* = 345)**		**KS noncase (2): Sym−/Dx+ (*n* = 106)**		**KS noncase (3): Sym−/Dx− (*n* = 239)**		**Severe GWI (*n* = 264)**		**Mild-to Moderate GWI (*n* = 665)**		**Noncase (*n* = 187)**	
	**Unadj %**	**Unadj %**	**Adj %**	**Unadj %**	**Adj %**	**Unadj %**	**Adj %**	**Unadj %**	**Adj %**	**Unadj %**	**Adj %**	**Unadj %**	**Adj %**	**Unadj %**	**Adj %**
PTSD	21.5	23.8	18.5	39.8	32.3	Sup.	Sup.	Sup.	Sup.	64.6	54.9	10.2	8.4	Sup.	Sup.
	(19.1, 24.1)	(19.8, 28.1)	(14.4, 22.6)	(34.6, 45.3)	(26.6,37.9)	Sup.	Sup.	Sup.	Sup.	(58.5, 70.4)	(47.3, 62.5)	(8.0, 12.8)	(6.1, 10.6)	Sup.	Sup.
Depression	28.6	32.5	28.8	51.3	46.9	Sup.	Sup.	Sup.	Sup.	79.5	76.4	16.2	15.3	Sup.	Sup.
	(26.0, 31.4)	(28.1, 37.2)	(24.1, 33.5)	(45.9, 56.8)	(40.9, 52.9)	Sup.	Sup.	Sup.	Sup.	(74.1, 84.3)	(70.4, 82.4)	(13.4, 19.2)	(12.4, 18.1)	Sup.	Sup.
Severe Pain	22.8	25.3	21. 6	40.0	33.4	Sup.	Sup.	Sup.	Sup.	60.3	53.0	13.5	12.5	Sup.	Sup.
	(20.4, 25.4)	(21.2, 29.7)	(17.3,25.8)	(34.8, 45.4)	(27.8, 39.0)	Sup.	Sup.	Sup.	Sup.	(54.1, 66.3)	(45.9, 60.1)	(11.0, 16.4)	(9.8, 15.2)	Sup.	Sup.

Note: All values were suppressed due to fewer than 11 people in cell sizes for (a) Sym−/Dx+, (b) Sym−/Dx−, and (c) CDC noncases. Chi square tests are all significant $p < 0.001$; for comparisons across groups; CDC = Centers for Disease Control and Prevention; Sup. = suppressed; Sym = symptom; Dx = diagnosis.

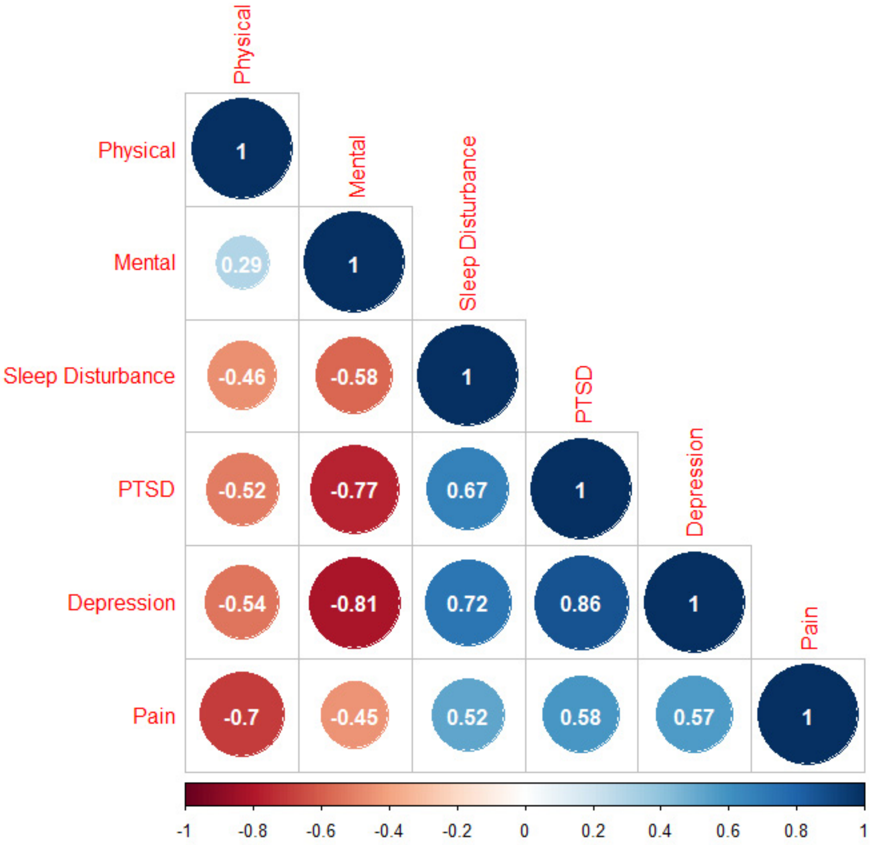

Figure S2. Association of mental and physical health related quality of life measures, depression, post-traumatic stress, sleep disturbance and pain, pairwise Pearson correlations; PTSD = post-traumatic stress disorder; All correlations statistically significant at $p < 0.001$.
